# Systematic gene function prediction from gene expression data by using a fuzzy nearest-cluster method

**DOI:** 10.1186/1471-2105-7-S4-S23

**Published:** 2006-12-12

**Authors:** Xiao-Li Li, Yin-Chet Tan, See-Kiong Ng

**Affiliations:** 1 Knowledge Discovery Department, Institute for Infocomm Research, 21 Heng Mui Keng Terrace, Singapore 119613, Singapore

## Abstract

**Background:**

Quantitative simultaneous monitoring of the expression levels of thousands of genes under various experimental conditions is now possible using microarray experiments. However, there are still gaps toward whole-genome functional annotation of genes using the gene expression data.

**Results:**

In this paper, we propose a novel technique called Fuzzy Nearest Clusters for genome-wide functional annotation of unclassified genes. The technique consists of two steps: an initial hierarchical clustering step to detect homogeneous co-expressed gene subgroups or clusters in each possibly heterogeneous functional class; followed by a classification step to predict the functional roles of the unclassified genes based on their corresponding similarities to the detected functional clusters.

**Conclusion:**

Our experimental results with yeast gene expression data showed that the proposed method can accurately predict the genes' functions, even those with multiple functional roles, and the prediction performance is most independent of the underlying heterogeneity of the complex functional classes, as compared to the other conventional gene function prediction approaches.

## Background

Recent emergence of various high throughput tools has supplied new and powerful means for biologists to experimentally interrogate living systems at the systems level instead of merely at the molecular level. Large-scale experiments that could only be imagined a few decades ago can now be performed routinely. In particular, the advent of DNA microarray technologies has enabled the differential expressions of thousands of genes under various experimental conditions to be monitored simultaneously and quantitatively. Analysis of such genome-wide gene expression data is useful for elucidating the functional relationships among genes in the genomes.

To systematically reveal the biological functional roles of the genes in a genome, the gene expression profiles of a series of experimental assays or conditions can be grouped into clusters based on the similarity in their patterns of expression. The co-expressed genes in each cluster can then be inferred to be coding for proteins that partake in a common biological function. The functions of unknown gene products can also be inferred using the guilt-by-association principle [1].

There are two typical techniques that can be used on gene expression data for gene function annotation or prediction. The first technique is clustering (a form of unsupervised learning), while the second is classification (a form of supervised learning) [2]. In clustering, the data points (e.g. genes) are unlabeled – in other words, we assume no prior knowledge about any of the genes' biological functions. Using the expectation that genes which perform a common biological function would have expression profiles that exhibit a similar pattern across different experimental conditions, the clustering process organizes genes into different functional groups using a similarity (or distance) measure on the gene expression data. Numerous clustering techniques [3] have been proposed to find groups of co-expressed genes. These techniques include hierarchical clustering [4], self-organizing maps [5], *k*-means clustering [6], simulated annealing [7], graph-theoretic clustering [8], mutual information approach [9], fuzzy *c*-means clustering [10], diametrical clustering [11], quantum clustering with singular value decomposition [12], bagged clustering [13] and CLICK [14].

Clustering techniques are useful when there is no prior knowledge (i.e. functional labels for the genes) available. However, this may not be a particularly common situation here as biologists typically already know a subset of genes involved in a biological pathway of interest. Instead of clustering, we can treat the function prediction problem as a classification task so that such prior information can be exploited in the form of training sets for supervised machine learning algorithms. Several classification methods have been proposed, including nearest neighbor classification [15], support vector machines [16] and neural networks [17]. However, as cellular functions are naturally complex, a combination of heterogeneous biological activities is typically required to perform each biological function. This means that not all the genes in a given functional class behave homogeneously, and this can drastically affect the learning rates of classification methods [17].

In this paper, we therefore adopt a combined approach of unsupervised clustering followed by supervised classification for assigning biological functions to the unknown genes. First, we perform hierarchical clustering to find co-expressed subgroups or clusters of genes within each putative heterogeneous functional class. After that, given a test gene, we predict its functional classes by computing the similarity of its expression profile to each of its nearest functional clusters – these similarity values can be considered as fuzzy membership values that represent the degree to which the test gene belongs to the corresponding functional classes (where each class is a fuzzy set). The function labels of those clusters with maximal similarities can then be assigned to the test gene as its predicted functions.

We call this approach the Fuzzy Nearest-Cluster method (FNC) and we will show in this paper that it is particularly useful for genome-wide systematic functional prediction of genes from microarray expression data, because it takes into account the heterogeneity present even within each functional class.

## Methods

In this section, we provide the details of our proposed technique Fuzzy Nearest-Cluster (FNC), which utilizes the advantages of both clustering and classification by (i) capturing the homogeneous gene subgroups within heterogeneous function classes through clustering; and (ii) using the experimentally-determined function information, i.e. prior biological knowledge for classification. Our method FNC consists of two steps. Section 'Mining for co-expressed gene subgroups with hierarchical clustering' presents the first step – a hierarchical clustering algorithm that finds, within each functional class, the subgroups of genes that are co-expressed. Then, a classification step is described in Section 'Predicting the functions of unclassified genes' to predict the functional classes of unclassified genes based on the functional similarities. Finally, Section 'Determining the thresholds λ and *k'* presents how to automatically set the parameters used in the two steps above.

### Mining for co-expressed gene subgroups with hierarchical clustering

**Figure 1:**
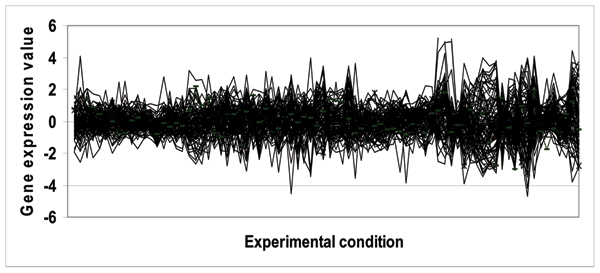
Heterogenous expressions of genes for the "C-compound and carbohydrate metabolism function" (MIPS code 01.05)

**Figure 2:**
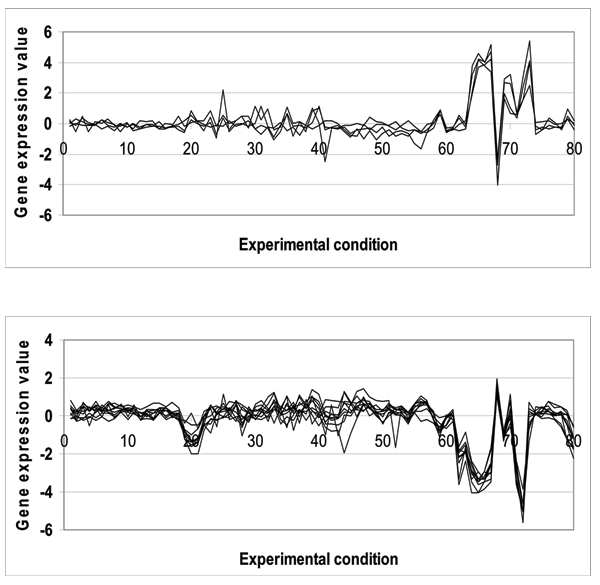
Two distinctly co-expressed sub-graphs for the genes in the "C-compound and carbohydrate metabolism" function.

Biological functions are complex processes; it is therefore unrealistic to expect that all the genes in a functional class would be expressed in a homogeneous fashion. Figure 1 shows an example of the high degree of heterogeneity amongst the genes in the functional class "C-compound and carbohydrate metabolism". It is thus desirable to capture the homogeneous gene subgroups within each functional class, where the genes within each subgroup have a maximal level of similarity in their expression (see Figure 2) that is in turn suitable for classification training. In this paper, we therefore pre-characterize each functional class by performing hierarchical clustering to group the genes within a given functional class into homogeneously co-expressed subgroups.

Agglomerative hierarchical clustering (HC) is an iterative procedure whereby the most similar genes are grouped together during each step to form progressively larger and larger clusters of genes. Compared with *k*-means clustering where the number of clusters must be pre-determined by a parameter *k*, the number of sub-clusters need not be pre-determined here (although HC typically clusters all the genes into one big cluster after the procedure is complete). It is therefore suitable for our application as it is not possible to pre-determine the number of subgroups in a heterogeneous functional class.

There are several approaches for agglomerative HC. In the average group linkage method, the distance (or inversely, similarity) between two clusters is defined in terms of the average vectors of each cluster, i.e. two vectors are involved. Other methods include average linkage (distance is average of pair-wise distances between all items within two clusters), single linkage (minimum of all pair-wise distances), and complete linkage (maximum of all pair-wise distances). Our FNC method employs a variant of average group linkage. We chose the average group linkage method (also used in [4]) for its computational efficiency as well as its robustness against the noisiness of gene expression microarray data. Single linkage and complete linkage are relatively much more susceptible to noise as they take only a single distance (either minimum or maximum) into account when comparing clusters.

Our variant of average group linkage produces a number of clusters instead one single cluster. We introduce a threshold λ to stop the clustering procedure when even the most similar or closest clusters have a similarity less than λ.

Algorithm 1 details our clustering algorithm for mining co-expressed subgroups within a functional class. For each function *f*_*i *_in the function set *F*, our Algorithm 1 clusters the genes within the functional group *f*_*i *_into co-expressed subgroups. In the algorithm, steps 3 to 6 construct a gene set *G*_*i *_for function *f*_*i *_and compute the corresponding similarity values between each pair of genes in the gene set *G*_*i*_. Here, the Pearson correlation is used as the similarity measure. In step 7, each gene in the *G*_*i *_is set as an initial individual cluster to construct cluster *C*_*i*_. Step 8 then finds the two most similar clusters from *C*_*i*_. Steps 9 to 15 comprise the merging loop to group the two most similar clusters into a new cluster *C*_*ik *_(step 10) if the similarity value is greater than the threshold λ. Step 11 then calculates the new expression profile for cluster *C*_*ik*_. Steps 12 and 13 add the new cluster while removing the two underlying clusters from *C*_*i *_respectively. Finally, step 14 finds the two most similar clusters in the updated cluster set *C*_*i *_to prepare for the next iteration. When the algorithm terminates, for each gene function *f*_*i*_, the algorithm outputs a cluster set *C*_*i *_where the similarity between each pair of clusters in *C*_*i *_is less than λ.

It is important to note that while genes were clustered together regardless of their biological functions in the related clustering works mentioned in the introduction, we cluster here only the genes within each of the functional classes. Thus, we are able to make use of existing biological knowledge and avoid the potential problem of generating gene expression clusters do not correspond to the true biological functional classes.

**Input**: Training gene set *G *and function set *F*

**Output**: Cluster set *C*_*i *_for each function *f*_*i*_

1: **BEGIN**

2: **for **each function *f*_*i*_∈*F ***do**

3: Construct gene set *G*_*i *_= {*g | fun*(*g*) = *f*_*i*_, *g*∈*G*};

4: **for **each pair of gene (*g*_*a*_*, g*_*b*_)*, g*_*a*_∈*G*_*i*_*, g*_*b*_∈*G*_*i*_, *a *≠ *b*, **do**

5: Compute the similarity *sim*(*g*_*a*_*, g*_*b*_);

6: **end for**

7: Initialize cluster set *C*_*i *_= {*C*_*ij*_*| C*_*ij *_= {*g*_*j*_}, *g*_*j*_∈*G*_*i*_, *j *= 1, 2, . . ., |*G*_*i*_*|*};

8: Find the two clusters *C*_*im *_and *C*_*in *_with maximal similarity,

(*C*_*im*_*, C*_*in*_) = *arg **sim*(*C*_*ia*_*, C*_*ib*_)*, C*_*ia*_*, C*_*ib*_∈*C*_*i*_;

9: **while **(*sim*(*C*_*im*_*, C*_*in*_) ≥ *λ*) **do**

10: Combine *C*_*im *_and *C*_*in *_into a bigger cluster *C*_*ik*_;

11: Calculate the expression profile for *C*_*ik *_by averaging the gene profiles of *C*_*im *_and *C*_*in*_;

12: *C*_*i *_= *C*_*i *_∪ {*C*_*ik*_};

13: *C*_*i *_= *C*_*i *_- {*C*_*im*_} - {*C*_*in*_};

14: Find the two new clusters *C*_*im *_and *C*_*in *_with maximal similarity in updated cluster set *C*_*i*_, *C*_*im*_*, C*_*in *_∈ *C*_*i*_;

15: **end while**

16: **end for**

17: **END**

**Algorithm 1. **Mining of co-expressed subgroups within each function

### Predicting the functions of unclassified genes

Next, based on the gene subgroups in each of the functional classes, we can predict the functions of unclassified genes by using their nearest clusters' functional information. The underlying rationale is that co-expressed genes are likely to share the same biological functions (the "guilt-by-association" principle). Given an unknown gene *g*_*t*_, for each function *f*_*i*_, we compute the functional similarity value between *g*_*t *_and *f*_*i*_. The gene *g*_*t *_is then assigned with functions having the largest similarity values. The function similarity value between g_*t *_and *f*_*i *_is computed as follows. First, we compute the Pearson similarity between *g*_*t *_and each cluster in function *f*_*i*_. The clusters that have the top *k *biggest Pearson similarity values are then selected as prototype clusters. The functional similarity between *g*_*t *_and *f*_*i *_is then defined as the average Pearson similarity value of the prototype clusters. The detailed steps are shown in Algorithm 2.

In Algorithm 2, we predict the functions for each unclassified gene *g*_*t *_in the test set *T *based on its similarity scores (also interpreted as a fuzzy membership value) with the clusters of the known functions. Step 4 of the algorithm computes the cluster similarity between a test gene *g*_*t *_and each cluster *C*_*ij *_in cluster set *C*_*i *_of function *f*_*i*_. Steps 5 to 6 then obtain a subset *C*_*top *_of *C*_*i *_consisting of *k *nearest prototype clusters. Step 7 computes the average cluster similarities *fs*_*i*_. Finally, steps 9 and 10 rank the *fs*_*i *_and assign the test gene *g*_*t *_with the functions that have the top *fs*_*i *_values (see our evaluation metric TNA in Section 3.1.3).

**Input**: Test gene set *T*, Cluster set *C*_*i *_for each function *f*_*i*_

**Output**: gene's predicted functions

1: **BEGIN**

2: **for **each test gene *g*_*t*_∈*T ***do**

3: **for **each function *f*_*i*_∈*F ***do**

4: Compute the cluster similarity *ss*(*g*_*t*_*, C*_*ij*_) between the test gene *g*_*t *_and each cluster *C*_*ij *_in cluster set *C*_*i*_;

5: Suppose cluster *C*_*ik *_is the cluster whose cluster similarity is *k*-th largest in cluster set *C*_*i*_;

6: *C*_*top *_= {*C*_*ij*_*| ss*(*g*_*t*_*, C*_*ij*_) *≥ ss*(*gt, C*_*ik*_)*, C*_*ij*_∈*C*_*i*_, *j *= 1, 2, . . ., |*C*_*i*_*|*};

7: 

8: **end for**

9: Rank *fs*_*i*_, *i *= 1, 2, . . ., |*F*|;

10: Assign the functions with the top *fs*_*i *_to gene *g*_*t*_;

11: **end for**

12: **END**

**Algorithm 2**. A fuzzy *k*-nearest clusters algorithm for functional prediction.

Given a test gene, the functions with maximal functional similarities will be assigned to it. The average cluster similarity *fs*_*i *_basically evaluates how similar a test gene is to a function, indicating a fuzzy membership value with respect to each function. The sum of fuzzy membership values for any particular test gene need not be 1, since these are not probability values. Also, because genes are typically involved in multiple cellular processes, each gene can have partial membership in more than one functional class (fuzzy set).

### Determining the thresholds λ and *k*

There were two parameters, λ and *k*, used in the two steps presented in the previous sections. λ is a parameter for the clustering process, while *k *is a parameter for the classification step. Parameter λ determines when we should stop the clustering process; its value directly affects the "quality" of the clusters output by the clustering step. Parameter *k *controls how many similar neighboring clusters to be used in the classification step for predicting the function labels for a given gene; it therefore affects the classification performance.

Conventionally, clustering and classification methods require the parameters to be "user-defined"; they therefore fall short for not providing a systematic way to determine the values for these key parameters that directly affect system performance. Here, we show how we can quantitatively determine the threshold values for these two parameters by minimizing the estimated error rate based on the known genes' function labels. We use different values of λ from 0.7 to 1.0 (in steps of 0.05) while varying *k *from 1 to 20 (with step 1.00). For each combination of λ and *k *values, we compute the estimated error rate for all the genes in training set *G *– by counting the number of genes' predicted functions *f*(*g*_*i*_, λ, *k*) that were not equal to its actual functions *L*(*g*_*i*_). The threshold values of λ and *k *can then be obtained from the (λ', *k*') that gave the minimum error on *G*:



## Results

Gene function prediction is a multi-class classification problem since genes typically play multiple roles biologically. Given an unclassified gene and multiple possible functional classes *C *= {*c*_*1*_, *c*_*2*_,..., *c*_*n*_}, our program needs to decide the most likely *N *classes for the unknown gene; the predictions can then be given to biologists for experimental validation. As such, we face a more challenging classification problem than typical binary classification that only needs to determine whether a gene belongs to a particular functional class or not.

### Experimental setup

For evaluation, we compare our proposed FNC method with two widely adopted methods, i.e. Support Vector Machines [18] and *k *nearest neighbors [15]. For each of the classification methods in our evaluation, we perform 5 randomly-seeded runs of 5-fold cross-validation.

#### Data set

We use a composite dataset from six different experimental studies described in [19,20] and [21]. Each study's dataset consists of gene expression levels of the entire yeast genome under various experimental conditions (see Table 1). Together, they form a composite dataset comprising the gene expression levels of 6221 genes under 80 different conditions. We represent the data as a matrix of 6221 rows and 80 columns. The composite dataset can be obtained from Eisen's lab [4] at .

Note that there are many missing values in the original 6221-by-80 data as some gene expression values were not obtained under certain conditions in the studies due to experimental limitations or irregularities. We further refine the dataset by filtering out those rows (genes) with more than 20 missing values, resulting in a reduction of classifiable genes to 5775. Some of these genes may still have missing expression values. Although there are various involved methods for filling in or predicting missing values [22], we simply fill in the missing values with zeroes here without loss of generality.

#### MIPS functional annotation

In our study, we use the MIPS Comprehensive Yeast Genome Database (CYGD) [23] as the source of function annotations. MIPS uses a numeric, hierarchical system to denote the various classes of biological functions. In this work, we use a functional granularity up to MIPS level 2. We then keep only those functional classes that contained at least ten genes so that there are sufficient training data for each function. In all, 48 MIPS functional classes were selected classifying the 5775 yeast genes using the 80-column datasets.

#### Evaluation metric

We introduce here a new evaluation metric called the "top *N *accuracy" (TNA). For each given gene, the TNA metric requires a prediction algorithm to produce a ranked ordering of all putative functional categories (there are 48 in the current case), in the order of decreasing likelihood for class membership. The algorithm is considered to have made a correct prediction if any of the *N *most likely classes is actually a function of the gene. The overall "top *N *accuracy" is then the percentage of test genes that are correctly predicted in this fashion. We set *N *= 4 here since in the MIPS system, a yeast gene typically has at most four different functions (only 2.3% of genes have 5 or more functions).

The TNA metric can be easily used on any algorithm whose outputs are continuous variables. For evaluation, it has numerous advantages over existing metrics such as accuracy, F-measure and cost-savings [16]. Compared to the traditional "accuracy" metric (used in [24]), TNA is more robust to unbalanced training sets (which is the present situation), where the negative examples outweigh positive examples by many times, such that a trivial algorithm that always returns a negative outcome will have a very high accuracy. Our TNA metric overcomes this by using a ranking system instead.

As compared to the "cost-savings" metric used in [16], TNA is more intuitive because it is similar to the familiar notion of accuracy. Also, the cost measure in [16] is defined as FP + (2 × FN), where FP and FN are the number of false positives and false negatives respectively. This formula not only makes the assumption that false negatives are twice as costly as false positives, it does not take into account the number of true positives and true negatives.

Furthermore, TNA is more intuitive and "usable" compared to F-measure, which is the harmonic mean of recall and precision. Having a combined metric that takes both recall and precision into account makes for easier comparisons, but lowers the interpretability of the results. For instance, what does an F-measure of 0.5 mean? In contrast, a TNA of 50% when *N *= 4 is easily and unambiguously interpreted to mean that given a set of genes, half of them will have at least 1 correctly predicted function among their top 4 predicted functions.

#### Compared techniques

As mentioned earlier, we compare our FNC technique with Support Vector Machines and *k *Nearest Neighbors.

Support Vector Machines (SVMs) [18] are a commonly used kernel-based machine learning technique for microarray data analysis. We use the SVMlight software  in our evaluation. Among the various possible kernel functions, we use the two popular kernels, the linear kernel and radial basis function (RBF) kernel, denoted as L-SVM and RBF-SVM respectively.

Note that SVMs perform binary classification; as such, we need to adapt it to perform multi-class classification for our purpose. To do so, we first trained 48 different binary SVMs, one for each function class. For prediction, each SVM outputs a real value (instead of a 1 or 0). Traditionally, a threshold of 0 is used to determine if the test sample is in the function class or not. Here, we compare the real values output by the 48 binary classifiers, and take the *N *predictions with the highest values. Note also that for RBF-SVM, the performance varies with 2 built-in parameters, γ and *c*. Parameter γ is the "width" of the RBF while *c *determines the trade-off between the training error and the width of the margin separating the positive and negative training examples. Both parameters were determined heuristically, using the "grid-search method" (i.e. systematically trying various {γ, *c*} pairs). In preliminary experiments, we found that varying the parameters exponentially (e.g. *c *= [1, 10, 100, 1000]) is a reasonable approach because performance is essentially unchanged over small changes in parameter values. We performed the grid-search at two levels of granularity, first finding a coarse interval that produces good results, and then searching within that interval.

*k *nearest neighbors (KNN) is another standard machine learning technique [15]. For a given gene, its *k *nearest neighbors are found, and the function class label possessed by the majority of these *k *neighbors is assigned to the gene. For *N *= 4, we use *k *= 14 to match the mean value of *k *for FNC. For multi-class predictions, the *N *most common labels among the *k *nearest neighbors are assigned to the unclassified gene.

### Experimental results

We compare the four different prediction techniques in terms of our evaluation metric TNA. Table 2 shows the detailed classification results of the 5 random runs (note that a 5-fold cross validation comparison is performed in each run) for the top 20 functional classes in size. The results show that our FNC method outperforms the other gene function prediction methods, obtaining a TNA value of 65.27%, which is 4.55%, 23.17%, and 4.76% higher than KNN, L-SVM, RBF-SVM respectively.

Compared with the other techniques, FNC consistently achieved the best prediction results, indicating that our method is suited for systematic gene function prediction to help biologists in their continuing search for the biological functions of genes. Furthermore, in terms of the computational processing time, the closest performing prediction method, RBF-SVM, required close to an order of magnitude more time than FNC.

Table 3 shows the overall comparison results of the different prediction techniques for all the 48 functional classes. Our FNC method outperformed with 22.11%, 3.85%, and 5.5% higher than the TNA values obtained by L-SVM, RBF-SVM, and KNN respectively, confirming that its superior results were not limited to the larger-sized functional classes.

We also investigate the performance of FNC with respect to two specific issues for gene function prediction on expression data: heterogeneity and multiple functions.

#### Heterogeneity

As mentioned earlier, there can be much inherent heterogeneity in the functional classes as biological processes are necessarily complex, carried out by gene and protein groups that perform various roles that contribute toward the overall biological functions (see Figures 1 and 2 for an example). We investigate whether the prediction methods are affected by the underlying heterogeneity in the expression data for each biological function. We use the heterogeneity measure as defined in [17] to quantify the degree of heterogeneity for different functional classes. The correlation of the prediction performance against the degree of heterogeneity in the functional classes is then computed for each prediction method. Based on our evaluation dataset, the Pearson correlations were -0.50, -0.53, -0.54, -0.64 for FNC, KNN, L-SVM and RBF-SVM respectively. The results showed that our method FNC is least correlated (hence, most robust) with the degree of the underlying heterogeneity in the functional classes.

#### Multi-function predictions

Biological functions are not stand-alone but inter-related cellular processes; as such, it is common for a gene to hold multiple functional roles. An important issue for gene function prediction is whether we can predict all the functions for those genes with multiple functions.

**Figure 3:**
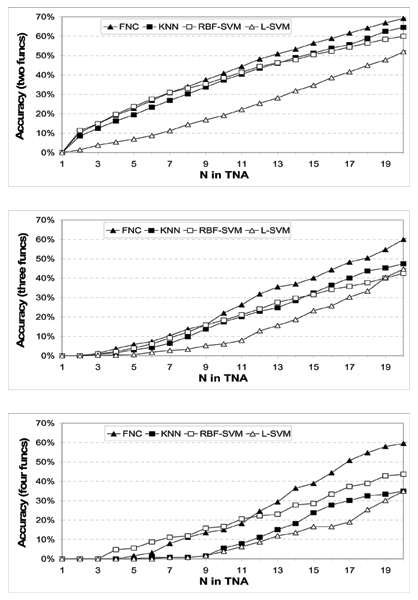
Comparison or results for genes with multiple (2, 3 and 4) functions (top to bottom respectively).

**Figure 4:**
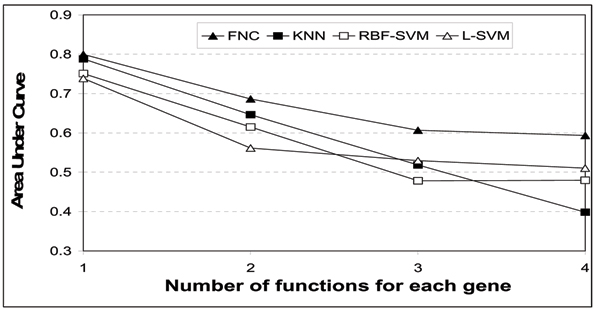
Comparison of areas under curves for genes with multiple functions.  Note that we use the full curves (up to N=48) for calculating the area, while Figure 3 shows the results for only up to N=20 due to space constraints.

Figure 3 shows the prediction results for genes with 2, 3 and 4 functions respectively. Here, we only show the results for up to the top 20 predictions (*N *≤ 20) due to space constraints. In all three cases, the prediction accuracy in terms of our TNA metric increases with *N*, as expected. Calculations of area-under-the-curve (where perfect performance gives an area of 1.0) confirmed that the ranking produced by our FNC method is consistently the best amongst all the methods (Figure 4). This means that our method FNC is more competent than the existing techniques in ranking the true functional classes in its top-ranked predictions. However, we should also note that there is still much room for improvement, as the accuracy values are still not high enough for small *N*.

## Conclusion

The recent advances in microarray technology have certainly revolutionized the way molecular biologists study the functional relationships among genes. While we are now able to monitor gene expression at the genomic scale using microarray technology, there are still gaps toward whole-genome functional annotation of genes using the gene expression data.

Gene function prediction is challenging because of several factors. For example, the larger functional classes are usually heterogeneous, while each gene in the genome can also play multiple functional roles. In this paper, we have described a robust Fuzzy Nearest-Cluster method for the systematic functional annotation of unclassified genes using DNA expression data. For each function, we do not assume homogeneity; instead, hierarchical clustering is first used to detect the homogeneous co-expressed subgroups for each functional class. This addresses the functional heterogeneity issue. Our FNC method then classifies the unknown genes based on their overall similarities to each detected functional clusters in a multi-class fashion. This addresses the possibilities of genes' playing multiple functional roles in the cellular processes. Our comprehensive comparative experimental results with yeast gene expression data showed that our method can accurately predict the genes' functions, even those with multiple functional roles, and at the same time, our method's prediction performance is also the most independent of the underlying heterogeneity of the complex functional classes, as compared to the other conventional gene function prediction approaches.

## Authors' contributions

XLL, YCT and SKN discussed and conceived of the algorithms. XLL designed the proposed techniques, performed analysis on the results and drafted the manuscript; YCT implemented the algorithms, performed analysis on the results; SKN supervised the project as a whole. All authors read and approved the final manuscript.

## Figures and Tables

**Table 1:** Experimental conditions in composite dataset

**Dataset**	**Type of condition**	**# conditions**	**Ref**
1	Nitrogen deficiency	13	[19]
2	Glucose depletion	7	[20]
3	Factor-based synchronization	18	[21]
4	Cdc15-based synchronization	25	[21]
5	Elutriation synchronization	14	[21]
6	Cln3 and Clb2 experiments	3	[21]

**Table 2:** Classification results (%) for largest 20 functional classes. Values in bold indicate the top performance in each row.

**Functional Class**	**FNC**	**KNN**	**L-SVM**	**RBF-SVM**
Mitochondrion	73.9	78.3	57.2	**78.7**
Cytoskeleton	69.7	**74.7**	46.7	61.3
Nucleotide metabolism	**39.4**	33.3	25.9	38.1
Protein targeting, sorting and translocation	**58.6**	48.6	40.0	47.7
Protein degradation	54.2	**54.6**	38.6	54.2
Cell growth/morphogenesis	67.5	**68.7**	44.4	59.7
Lipid, fatty acid and isoprenoid metabolism	31.5	29.9	29.3	**34.4**
Stress response	57.2	**58.7**	36.9	55.0
Amino acid metabolism	53.1	43.6	41.0	**57.3**
Cellular sensing and response	**63.1**	62.7	47.8	56.8
Protein modification	44.1	39.5	35.3	**47.3**
Ribosome biogenesis	90.0	**94.5**	84.8	94.1
RNA processing	**50.7**	48.4	31.6	47.7
DNA processing	**71.0**	63.1	39.5	64.7
Transported compounds	**73.8**	60.4	36.8	68.7
Fungal/microorganismic cell type differentiation	73.5	**76.2**	45.6	66.0
C-compound and carbohydrate metabolism	**76.3**	63.9	41.2	69.7
Cell cycle	**86.5**	79.1	44.3	76.0
RNA synthesis	**83.1**	64.3	33.7	66.5
Transport routes	**88.3**	72.1	41.4	66.1

**Average**	**65.27**	60.72	42.10	60.51

**Table 3:** Comparison of results (%) of whole-genome functional classification. Values are derived from the mean of 5 random repetitions of 5-fold cross-validation.

**Method**	**Run 1**	**Run 2**	**Run 3**	**Run 4**	**Run 5**	**Mean**
L-SVM	35.30	34.82	34.02	34.02	34.08	**34.45**
RBF-SVM	53.20	53.20	51.80	52.44	52.92	**52.71**
KNN	50.90	50.98	51.14	51.54	50.74	**51.06**
FNC	56.76	56.52	56.02	56.98	56.50	**56.56**
